# Anterior cingulate cortex: A brain system necessary for learning to reward others?

**DOI:** 10.1371/journal.pbio.3000735

**Published:** 2020-06-12

**Authors:** Patricia L. Lockwood, Kathryn C. O’Nell, Matthew A. J. Apps

**Affiliations:** 1 Department of Experimental Psychology, University of Oxford, Oxford, United Kingdom; 2 Wellcome Centre for Integrative Neuroimaging, University of Oxford, Oxford, United Kingdom; 3 Centre for Human Brain Health, School of Psychology, University of Birmingham, Birmingham, United Kingdom

## Abstract

Helping a friend move house, donating to charity, volunteering assistance during a crisis. Humans and other species alike regularly undertake prosocial behaviors—actions that benefit others without necessarily helping ourselves. But how does the brain learn what acts are prosocial? Basile and colleagues show that removal of the anterior cingulate cortex (ACC) prevents monkeys from learning what actions are prosocial but does not stop them carrying out previously learned prosocial behaviors. This highlights that the ability to learn what actions are prosocial and choosing to perform helpful acts may be distinct cognitive processes, with only the former depending on ACC.

## Abstract 2

How do we learn which actions benefit others? This Primer discusses a recent study showing that removal of the anterior cingulate cortex prevents monkeys from learning new actions that benefit others, but does not stop them from carrying out previously learned prosocial acts.

Prosocial behaviors—actions that help others, often without regard to whether they directly benefit us—are vital for social cohesion. From a stranger holding an elevator door to the millions of charity volunteers worldwide, humans readily engage in a range of helping behaviors. Other species can be prosocial too, such as when a monkey grooms another member of its troop. Understanding the psychological processes and brain systems that underlie how one individual decides whether to help another out has become a major endeavor in cognitive and behavioral neuroscience [1,2]. The aim is to identify brain areas that perform cognitive processes that result in a decision to help others and not just do actions that directly benefit ourselves. In this field, one of the key questions that remains unanswered is: how do we learn what actions are prosocial?

One recent, striking conceptual advance is the introduction of the notion of prosocial or “vicarious” learning [3,4] (Fig 1). The idea is that in order to do acts that benefit other people, we first have to learn what actions in the world will lead to something rewarding and positive happening to them. When we perform an action, and it results in a reward for another person, we can put ourselves in their shoes and imagine vicariously what it would be like to get a similar reward ourselves. For example, imagine you see a notebook fall out of someone’s bag in front of you, you pick it up, give it back to them, and they smile and thank you. Vicarious processing of the benefit to the other person (the relief of having not lost their notebook), may be part of the foundations of how we learn to do prosocial acts and how we adapt our prosocial behavior in different settings [5,6]. Theoretically, this is important, because it means that some people may fail to be prosocial not because they have nefarious intentions but because they fail to learn what acts will benefit or harm others.

**Fig 1:**
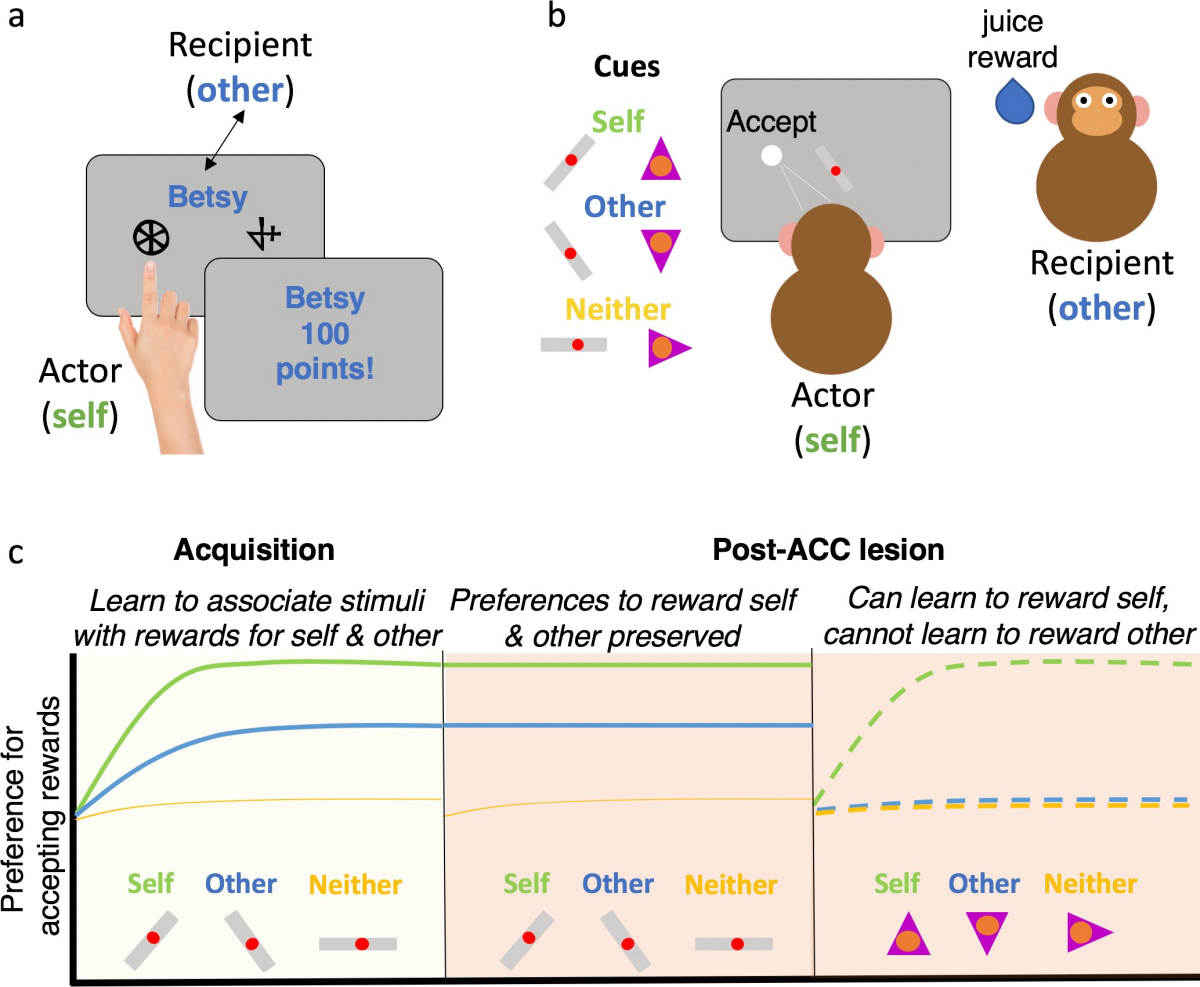
Prosocial learning tasks in humans and monkeys. (a) Example of the prosocial learning task used in humans [4]. Participants learn by trial and error which of 2 pictures is more likely to deliver a reward. One picture has a high probability of reward, and one has a low probability of a reward. On some blocks and trials, participants play for outcomes for themselves (Self), on some, they play for outcomes for another person (“Betsy” in this example), and on some trials, neither person receives the reward (no one). (b) Example of the prosocial learning task used by Basile and colleagues (2020) [15]. Monkeys learned the outcomes of 3 different cues, some cues rewarded only the monkey themselves (self), some cues rewarded the social partner (other), and some cues rewarded neither monkey (neither). Monkeys first fixated on a cue in the of the screen, and then a target appeared that they had to saccade to if they wanted to accept the offer or break fixation if they wanted to reject the offer. (c) Schematic of the results of Basile and colleagues. Before lesions to the ACC, monkeys were able to learn which cues rewarded themselves, other, or neither and showed a preference for “self over other” and “other over neither.” After ACC lesions, monkeys were able to learn with a new set of cues which cue rewarded themselves but could no longer learn to associate stimuli with other or neither. ACC, anterior cingulate cortex.

The computations that underlie such learning may well parallel those that guide how we learn for ourselves. Reinforcement learning theory (RLT) is a powerful mathematical framework for how we do this type of learning and has been widely applied in many areas of psychology and neuroscience [7,8]. The key idea in RLT is that learning is driven by “prediction errors” (PEs). When you perform an action, you make a prediction about how good the outcome will be. If the outcome is surprising and better or worse than you expected, how wrong your expectation is dictates the size of your PE. PEs drive learning by updating our future predictions of how good an action is. If the outcome is better than we expected (a positive PE), it increases our expectations that picking that action will result in a good outcome, and we become more willing to choose it. When the outcome is worse than expected (negative PE), we update our expectations and are less willing to choose that action.

RLT has been deployed to understand the vicarious processing of others’ rewards [2,9–13]. Understanding how the brain does this is seen as crucial, as it may underpin prosocial learning. Brain imaging studies in humans have measured changes in the blood-oxygen-level-dependent (BOLD) signal—a proxy of neural activity—when people are performing a variety of different tasks that require making predictions or processing rewarding outcomes during social interactions. Activity in the brain is correlated with the magnitude of rewards and the size of PEs when those outcomes are delivered to other people [2,9–11]. Similarly, it has been shown that individual neurons in the brain correlate with PEs when people are watching and learning from the outcomes of others’ decisions [14].

These social PE and reward signals had been identified in several brain regions, but considerable evidence pointed to their presence in parts of the anterior cingulate cortex (ACC), a brain area lying on the medial surface of the frontal cortex [2,6,10] (Fig 2). It had therefore been suggested that this region might be important for prosocial behavior and learning. However, although the recordings from individual brain cells or activity in brain areas are very useful, they only provide correlational evidence between brain responses and prosocial behavior. They cannot tell us if these brain areas are necessary for being prosocial. That is, without this brain area, would people stop choosing or learning to be prosocial?

**Fig 2:**
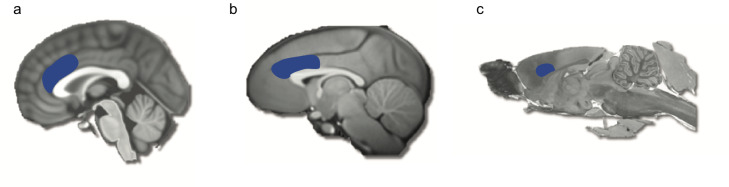
**Anatomical location of the anterior cingulate cortex in the human (a), macaque (b), and rat (c) brain.** This region has been linked to prosocial behavior and vicarious reward processing across species [9–10, 15–16]. The blue area was lesioned in Basile and colleagues, with the approximately equivalent anatomical zones highlighted in the other species.

Basile and colleagues provided an important step in testing whether ACC was necessary to learn what will benefit others [15]. They had 6 macaque monkeys take part in an experiment that allowed them to learn and execute prosocial choices and then performed a lesion—removing brain tissue—in the ACC (Fig 2), to see whether it impacted behavior. The monkeys performed a task in which rewards (juice) could be delivered to themselves (self), they could observe another monkey (other) receiving a reward, or neither monkey would get a reward (neither). The monkeys first had to learn which of a set of abstract pictures were associated with rewards for self, other, and neither (Fig 1). Once this had been learned, they performed a decision-making task, in which they were able to accept or reject an offer to deliver a reward across 3 conditions (self, other or neither). Much like humans, the monkeys showed an apparent selfishness, accepting rewards more often for self compared with other or neither [3,4,16]. However, when comparing acceptance of rewards for other and neither, monkeys rewarded the other more frequently (Fig 1). Thus, with an intact ACC, monkeys could learn what stimuli were predictive of giving others rewards, and they chose it—they learned what actions were prosocial, and they had a preference to be prosocial.

Next, they lesioned portions of the sulcus and gyrus of the ACC in half of the monkeys. This ACC region contains distinct “zones” which have different cellular properties, that are referred to as areas 24 and 32 (Fig 2) [17]. The monkeys then performed the decision-making task again, with the same pictures as before, but also a new set of pictures, which were similarly associated with rewards being delivered to self, other, or neither. With the previously used pictures, the monkeys continued to show some evidence of a prosocial preference, picking to reward others more than neither. Importantly, the monkeys were also able to learn which pictures would get themselves rewards. So, their prosocial preferences did not depend on an intact ACC, and neither did self-related learning. However, there was a striking change in behavior: The monkeys did not learn that a new picture was associated with giving a reward to the other monkey. They could not learn to be prosocial after an ACC lesion (Fig 1).

These results suggest that learning what acts are prosocial and someone’s preference for being prosocial are distinct. The fact that one process is spared after a lesion while the other is disrupted indicates that they rely on separable cognitive processes, and only prosocial learning depends on the ACC. This is important, as few studies in human or nonhuman primates had examined a causal link to any brain area in giving a reward to others [2,10]. Establishing the necessity of a brain area is not straightforward. In humans, for valid ethical reasons, one cannot perform circumscribed lesions for experimental purposes. Moreover, widely used brain stimulation techniques that can provide causal evidence in humans are not currently able to target structures that lie deep below the surface of the brain, such as the ACC.

Intriguingly, recent research in rodents offers evidence that is consistent with the necessity of ACC for prosocial behavior. In rodent area 24 (Fig 2), which has been argued to be similar to primate ACC area 24 [17], neurons have been shown to respond when another rodent is seen in distress, and inactivating these neurons leads to a reduced ability of rodents to learn from the observation of another’s distress [18–21]. Thus, across species, the ACC appears to be necessary for learning from the outcomes of events that impact on others. Although it can be challenging to translate findings because of potential differences in the anatomical and functional properties of brain areas across species (such as differences in how strongly connected a brain area is to others) and differences in methodology, it is notable that the ACC is similarly implicated in processing social information across species. It would be interesting for future rodent studies to examine the role of the ACC in giving rewards to others in the manner monkeys and humans seem willing to do.

Although the findings of Basile and colleagues are exciting, a limitation of the work was the size of the lesion. The lesion covered both the sulcus and gyrus of the ACC, areas 24 and 32. However, previous work had suggested that the locus of social processing might be narrower; some work highlights a bigger contribution of area 24 than area 32 [10,20], and other studies highlight a bigger contribution of ACC gyrus than the sulcus [9,10,22]. Both of these might be true, with social processing specialized to the gyral portion of area 24[10,11]. Lesions to the gyrus lead to monkeys being less distracted by pictures of other monkeys, whereas lesions to the sulcus have no such effect [22]. Moreover, brain imaging studies in humans and electrical recording studies in monkeys have shown that ACC gyrus signals information about others that it does not signal about ourselves [10,11]. This includes predictions about others’ rewards and PEs when others’ outcomes are surprising [9–11]. In contrast, the ACC sulcus processes similar information about self and other [10,11]. Future work can address this limitation by making more specific lesions to examine the relative contributions of different subregions to prosocial learning.

Another potential avenue for research is to examine whether the ACC is involved in learning how to be prosocial through vicariously experiencing others’ outcomes or whether its function is about learning social information at a simpler level. One recent brain imaging study in humans found that activity in the gyrus of the ACC signaled a PE when learning that a stranger’s name was associated with an abstract picture but not when learning that one’s own name was associated with a different abstract picture [23]. It could be that the ACC guides this much more rudimentary form of learning. In particular, it raises the possibility that study by Basile and colleagues [15] the monkeys could not learn that the new pictures were associated with the other monkey after lesions, and it was this process of learning new associations about others that was disrupted, rather than vicarious processing of the reward per se. Future work disentangling learning simple associations between others and stimuli from those involved in explicitly learning what behaviors reward others will allow us to better understand prosocial behavior and the function of the ACC.

The findings of Basile and colleagues could potentially offer new insights into understanding impairments to social cognition. Many studies have linked disruptions of ACC functioning to atypical social behaviors across psychiatric conditions, particularly autism spectrum disorders (ASD) [10,11]. For example, some correlational evidence had shown that atypical PE signaling when vicariously processing others’ rewards is related to the ACC in those with ASD [24]. Basile and colleagues provide an important link to show that, indeed, without signaling in the ACC, monkeys cannot learn to obtain rewards for others, strengthening the claim that aberrant signaling in this region in ASD may be causally linked to social reward–processing deficits. Moreover, it also highlights that people with an ASD may have the intention to be prosocial, but failures to do so could be due to an inability to appropriately learn what actions will benefit others—a novel hypothesis that can be examined in future studies.

Understanding the psychology and neuroscience of prosocial behaviors is a multidisciplinary effort, requiring diverse approaches, of which causal evidence in primates is vital but sparse. Basile and colleagues offer an important window into this, dissociating learning from one’s intentions to be prosocial and linking it to ACC integrity. Future work can build on this robust causal evidence to understand the mechanisms across species and links to disrupted social behavior.
